# Premature Burial and the Law

**Published:** 1912-04-20

**Authors:** H. R. Gawen Gogay

**Affiliations:** 7 Baxter Avenue, Southend-on-Sea.


Sir,—Mr. Alfred E. Barrett, M.R.C.S., judging from the papers, is evidently much impressed with the fact that the community is running a very real risk of not only being buried alive when only in a trance or cataleptic state, but of being murdered also without risk of discovery, seeing that "the law is satisfied by the doctor certifying that somebody has informed him that his patient is dead"; and then Dr. Barrett asks, "Why should the doctor trouble himself any further about it?" There is every reason why the doctor should trouble himself; but until the law permits him to charge for the certificate poor humanity must take the risk of either being buried in a trance or of being murdered by Act of Parliament. Seeing the terrible danger the public are in from these two possibilities, the British Medical Council should approach the Government on the subject, with a view to a Bill being introduced and passed as quickly as possible.—Yours truly, H. R. Gawen Gogay.

7 Baxter Avenue, Southend-on-Sea.

